# Abnormal Blood Biomarkers and Cumulative Disability Burden in Middle-Aged and Older Adults: Evidence from Two Nationally Representative Surveys in the United States and China

**DOI:** 10.3390/jcdd12110429

**Published:** 2025-10-31

**Authors:** Raoping Tu, Jin-Jing Pei, Alexander Wolthon, Yueping Li, Hui-Xin Wang

**Affiliations:** 1 School of Health Management, Fujian Medical University, Fuzhou 350122, China; 2 Division of Psychobiology and Epidemiology, Department of Psychology, Stockholm University, SE-114 19 Stockholm, Sweden; jin-jing.pei@su.se (J.-J.P.); alexander.wolthon@ki.se (A.W.); 3 Department of Comparative Medicine, Karolinska Institutet, SE-171 77 Stockholm, Sweden; 4 Fujian Medical University Library, Fuzhou 350122, China; fmulyp@163.com; 5 Department of Clinical Neuroscience, Karolinska Institutet, SE-171 77 Stockholm, Sweden

**Keywords:** blood biomarker, biological system, disability, cohort study

## Abstract

Background: Few studies have simultaneously examined how blood biomarkers for inflammation, metabolic, and cardiovascular function are associated with disability incidence. This study aimed to comprehensively examine these associations. Methods: We used data from adults aged 50 and older in the Health and Retirement Study (*n* = 9250) and the China Health and Retirement Longitudinal Study (*n* = 6844), with biennial follow-up over a 4-year period. We defined abnormal biomarker values using standard clinical cut-off points for three biological systems. Disability burden was quantified as the cumulative number of impairments in basic and instrumental activities of daily living. Multivariate linear mixed-effects models were used to analyze the associations. Results: At baseline, 42% of participants had abnormal biomarker values in at least one system, 28% in two systems, and 7% in all three. A dose–response relationship was observed between the rate of disability accumulation and the number of systems with abnormal biomarker values. Compared to individuals with normal values across all systems, those with abnormalities in two systems had a significantly faster annual increase in disability burden (β = 0.06, 95% CI: 0.02–0.09), while those with abnormalities in all three systems exhibited an even steeper increase (β = 0.1, 95% CI: 0.05–0.16). Conclusions: The presence of abnormal levels in any two or all three of the systems significantly accelerated the rate of disability accumulation over a 4-year period. These findings highlight the importance of integrated biomarker monitoring for early identification of individuals at risk and inform the development of targeted preventive strategies.

## 1. Introduction

Given that approximately 31% of adults aged 50 years and older experience disability [1] and that the aging population continues to grow, it is essential to identify key pathophysiological factors that can guide interventions aimed at improving older adults’ physical function and quality of life.

Despite a growing interest in identifying blood biomarkers for disability, most studies have focused on a single blood biomarker [2,3,4] or an index of physiologic biomarkers [5,6,7], leaving the associations between blood biomarkers and disability less clearly understood. The accumulation of chronic stress and life events has been recognized as a source of allostatic load, which is identified by the use of biomarkers and clinical criteria, including the mutual effect between various physiological systems and their different levels of activity [8]. According to the allostatic load (AL) framework [9], biological systems can be represented by one or a combination of multiple blood biomarkers. For example, the metabolic system can be represented by high-density lipoprotein cholesterol (HDL-C) and glycosylated hemoglobin (HbA1c), and the cardiovascular system by systolic and diastolic blood pressure (SBP, DBP) and heart rate.

Different combinations of biomarkers included in the AL framework have been used in various studies. For example, by selecting items from the above battery, cross-sectional associations have been demonstrated between dysregulations of multiple biological systems and intrinsic capacity in Costa Rica [10], mobility disability in Brazil [11], ADL disabilities in Taiwan [12,13], frailty in Canada [14], and physical performance in North and South America [15]. These findings were confirmed in relation to mortality [16] and frailty in prospective community-based cohort studies consisting of high-functioning Americans [17] and middle-aged to older adults in the United Kingdom [18], as well as to the incidence of ADL and/or IADL disability in Boston Puerto Ricans [19]. Further, using combinations of different biomarkers without considering the biological system, longitudinal associations have been reported in relation to frailty in a nationwide prospective cohort in England [20]. In addition, some studies reported a dose–response association between the number of affected biological systems and intrinsic capacity [10], ADL impairment [13], frailty [17]. However, only one study focused on disability incidence [19].

The application of blood biomarkers offers an opportunity to reduce disease heterogeneity in clinical trials and epidemiological studies, and to gain a better understanding of the natural history of diseases [21]. However, most studies have used combinations of multiple biomarkers to represent each biological system in relation to disability. To our knowledge, no study has utilized a single biomarker or minimal set of biomarkers to depict individual biological systems. Moreover, the relationship between multiple biological systems and disability incidence over time has not been addressed in both the U.S. and Chinese cohorts and compared across the two nations.

The aim of this study was to systematically investigate the associations between abnormal blood biomarker levels related to inflammation, cardiovascular health, and metabolic function, depicted by a minimal set of biomarkers and self-reported hypertension or use of antihypertensive medication, and disability accumulation among individuals aged 50 and older within both U.S. and Chinese cohorts

## 2. Materials and Methods

### 2.1. Study Population

#### 2.1.1. The Health and Retirement Study (HRS)

**Figure 1:**
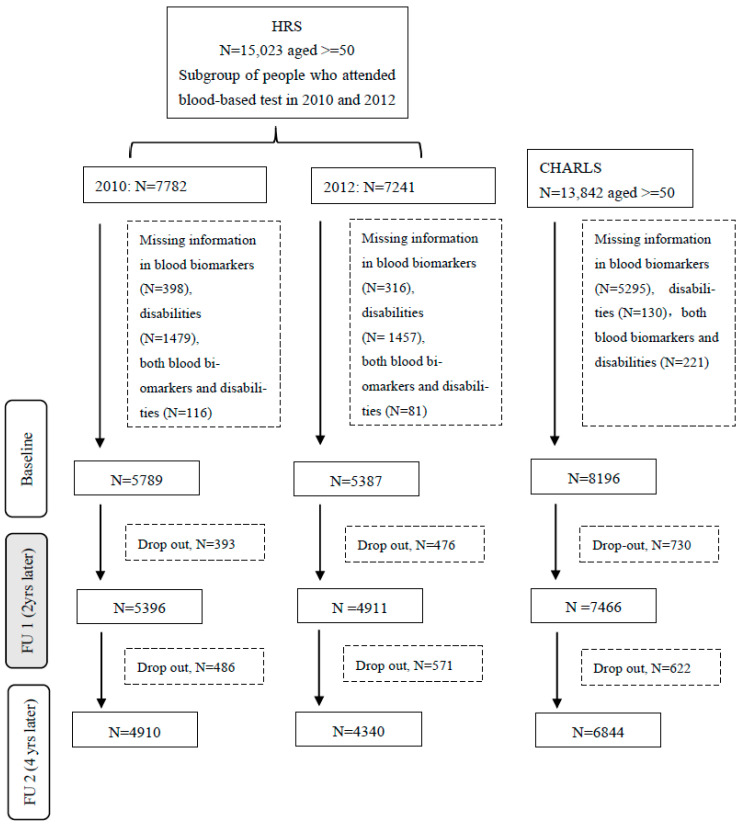
Flowchart of the study populations in HRS and CHARLS. Drop-outs are due to death, incomplete assessment, loss to follow-up or refusal in HRS and CHARLS. HRS, Health and Retirement Study; CHARLS, China Health and Retirement Longitudinal Study; FU 1, follow-up 1; FU 2, follow-up 2.

The sample was recruited based on a multistage area probability design. In 2006, a random half-sample of HRS households was selected for a face-to-face interview, with blood biomarker data subsequently collected during 2010–2014. The remaining half sample was interviewed in 2008, with blood biomarker data collected during 2012–2016 [22]. Participants with missing information on blood biomarkers or disabilities data, or those who dropped out after the baseline assessment, were excluded, leaving 9250 individuals in the analyses (Figure 1).

#### 2.1.2. The China Health and Retirement Longitudinal Study (CHARLS)

The initial sample was recruited using a stratified multistage probability sampling method in 2011 and has been followed biennially. Baseline blood samples were collected every second follow-up survey [23]. Data from the 2011 and 2015 surveys were used due to the availability of blood samples. Among the 17,705 participants in 2011, those younger than 50 years were excluded to match the HRS age range. Participants with missing information on blood biomarkers or disability data, and those who dropped out after the baseline survey, were excluded, leaving 6844 people in the analytical sample (Figure 1).

### 2.2. Defining Abnormal Levels of Blood Biomarkers in Different Biological Systems

We selected CRP, HDL-C, and SBP/DBP as biomarkers under the allostatic load framework [16] that are available in both the HRS and CHARLS cohorts to represent three key biological systems (inflammatory, metabolic, and cardiovascular). Rather than constructing a composite AL index, we applied the AL concept to guide the biomarker section. These biomarkers were chosen because they are reliably measured, available in both datasets, and biologically relevant. Other commonly used biomarkers of metabolic and cardiovascular health were not included, such as triglycerides and LDL-C, which were not measured in HRS; and HbA1c, which was excluded due to its higher assay cost and technical complexity compared with HDL-C. Utilizing this minimal biomarker set balances scientific rigor, feasibility, cost-effectiveness, and interpretability. Based on established cutoffs, these biomarkers were operationalized as follows: (1) inflammation represented by CRP > 3 mg/L [24], (2) metabolic dysfunction by HDL-C < 40 mg/dL in males, HDL-C < 50 mg/dL in females [25], and (3) cardiovascular anomalies by SBP ≥ 140 mmHg or DBP ≥ 90 mmHg, or self-reported hypertension or use of antihypertensive medication.

HRS: Finger-prick dried blood spot (DBS) samples were collected by trained interviewers at baseline (2010/2012). The capillary blood CRP was measured with enzyme-linked immunosorbent assays (ELISAs), and HDL-C levels were assessed by fluorometric assays. SBP and DBP were each recorded three times using an automated blood pressure monitor [26].

CHARLS: Venous blood was collected by trained medical staffs at baseline (2011) under a standard protocol developed by the Chinese Center for Disease Control and Prevention (China CDC). CRP was determined by immunoturbidimetric assays, and HDL-C by enzymatic colorimetric test. SBP and DBP were measured three times using an electronic sphygmomanometer [27].

Given the different blood collection and assaying protocols between the cohorts (DBS in HRS vs. venous blood in CHARLS), we considered the potential for measurement variation. A methodological study by Crimmins et al. [28] demonstrated that while DBS-based CRP values are highly correlated with venous measures, cholesterol measures (including HDL-C) from DBS are less reliable. To mitigate measurement bias and enhance comparability between cohorts, we employed standardized clinical cut-offs, allowing analyses to focus on clinical risk categories rather than absolute biomarker value and reducing sensitivity to systematic differences in measurement methods.

### 2.3. Assessment of Disability

In the HRS, ADL included dressing, eating, using the toilet, bathing, showering, getting in and out of bed, and walking across a room. IADL included preparing a hot meal, shopping for groceries, taking medications, managing their money, and making telephone calls. In the CHARLS, walking across a room in the ADL was replaced by controlling urination and defecation, while making telephone calls in the IADL was replaced by doing household chores. The numbers of difficulties in the ADL and IADL was first calculated separately and then summed to create a total difficulty score (ranging from 0 to 11) to increase the sensitivity of measurement [29].

### 2.4. Covariates

Age, sex, education, marital status, smoking, alcohol consumption, BMI, health status, and depressive symptoms were included as covariates. Maximal years of education achieved were dichotomized as follows: ≤9 years and >9 years, marital status as married and unmarried, smoking as current and non-current smokers (including former smokers), and alcohol consumption as regular alcohol drinkers (more than three times per week) and irregular alcohol drinkers. Body mass index (BMI) was calculated as weight (kg) divided by height squared (m^2^), and categorized as underweight (<18.5), normal weight (18.5–24.99), overweight (25–29.99), and obese (≥30).

Health status was determined by self-report of any doctor diagnosed chronic disease. Depressive symptoms were assessed using the 8-item Center for Epidemiological Studies Depression scale (CES-D) in HRS and the 10-item version in CHARLS, respectively, and were dichotomized as ≥4 in the HRS and ≥10 in the CHARLS, which are equivalent to the conventional cut point of ≥16 on the full 20-item CES-D [30,31].

All covariates were analyzed as baseline measures. Age was treated as a continuous variable, while all other covariates were treated as categorical in the statistical models.

### 2.5. Statistical Analysis

First, the distribution of normal and abnormal biological systems in one, two, or all three systems was compared between the U.S. and the Chinese cohorts. Analysis of variance (ANOVA) and chi-squared tests were used for continuous and categorical variables, respectively. Second, the proportions of individuals with normal/abnormal levels of blood biomarkers in each biological system were calculated in the pooled dataset (HRS + CHARLS). Third, the associations between different combinations of biological systems and the rate of disability accumulation (i.e., the number of disabilities over 4 years) were analyzed using linear mixed-effects models. These models extend simple linear models by incorporating fixed and random effects to account for within-person correlation due to repeated measures. Fixed effects included the number of dysregulated biological systems, follow-up year, and their interactions, while random intercepts and slopes captured individual differences in baseline disability and in the rate of disability accumulation over time. Finally, stratified analyses by education were performed to examine the potential interaction between education and biomarkers on ADL/IADL disabilities, given prior evidence linking education to ADL/IADL disabilities [32].

### 2.6. Sensitivity Analysis

Four sets of sensitivity analyses were conducted to test the robustness of findings by repeating the main analyses: (1) Excluding individuals with any ADL or IADL disabilities at baseline (*n* = 4064) to minimize reverse causation; (2) using an alternative clinical cutoffs: HDL-C < 40 mg/dL to test the robustness of metabolic classification; (3) including individuals with missing biomarker data (with missing values imputed using multiple imputation by chained equations) to assess bias due to missingness; (4) analyzing the HRS and CHARLS cohorts separately to evaluate cohort-specific associations.

All statistical analyses were performed with Stata SE 18.0 (College Station, TX, USA).

## 3. Results

### 3.1. Characteristics of the Participants

Descriptive data for the two national samples and categories of normal and abnormal biological systems are presented in Table 1. In both the U.S. and the Chinese samples, individuals with a higher number of abnormal biological systems tended to be older, female, unmarried, irregular alcohol drinkers, obese, and to have worse health status as well as more ADL and IADL disabilities. However, the U.S. adults with a higher number of abnormal biological systems were more likely to have lower levels of education, more depressive symptoms, and to be current smokers, whereas Chinese adults with a higher number of abnormal biological systems had similar level of education and depressive symptoms but were less likely to be current smokers compared with those with normal biomarker levels. Individuals with missing values for biomarkers and/or disabilities in the pooled dataset were more likely to have normal weight and better health status compared with those with complete information (Supplementary materials Table S1).

The proportions of individuals with normal and abnormal values in each biological system category are shown Figure S1. Overall, 23% of the total sample had normal values across all biological systems, while 42%, 28% and 7% had abnormal values in one, two, and three biological systems, respectively.

### 3.2. Blood Biomarkers and Disabilities

**Figure 2:**
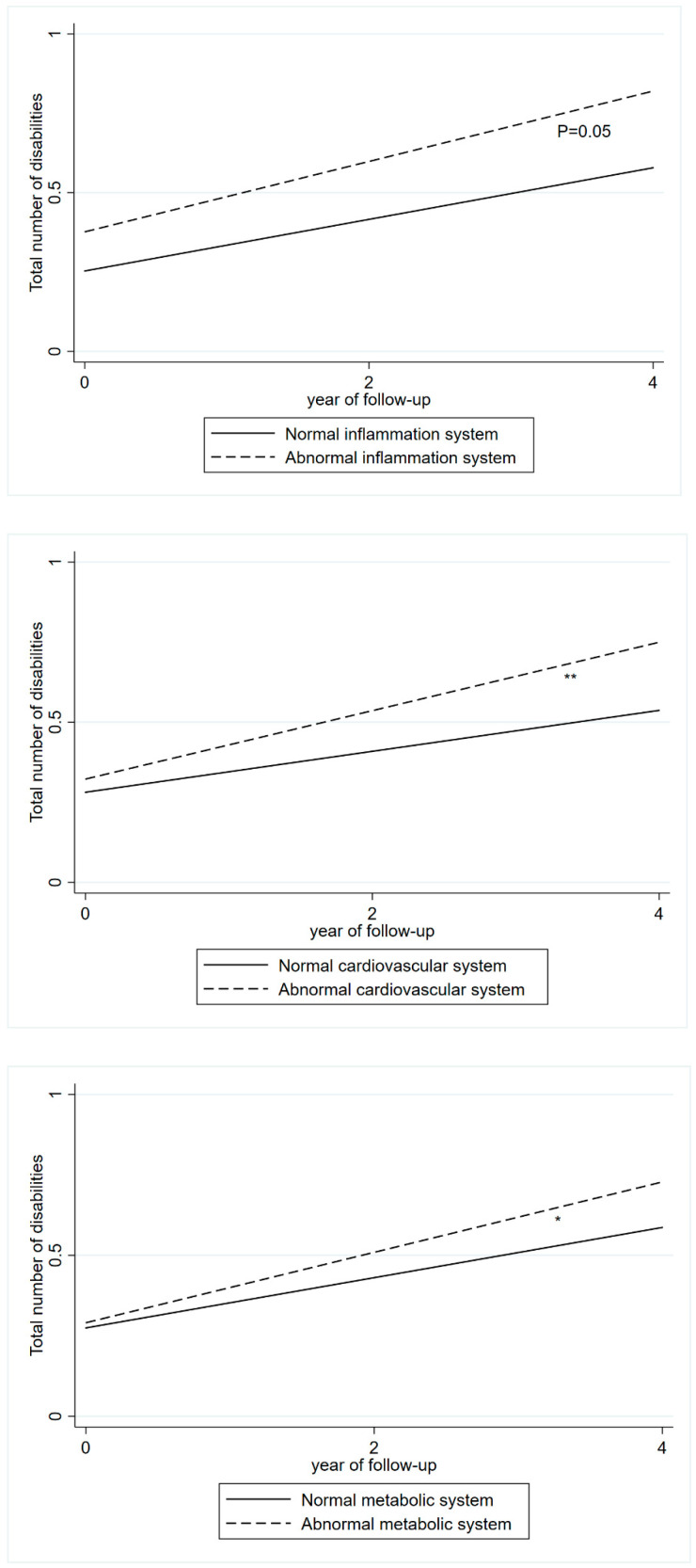
The cumulative burden of disabilities associated with the baseline abnormal levels of blood biomarkers in one biological system. The cumulative burden was derived from linear mixed-effects model adjusted for age, sex, education, marital status, smoking, alcohol consumption, BMI, health status, and depressive symptom. Abnormal inflammation system: CRP > 3mg/L. Abnormal metabolic system: HDL-C < 40mg/dL in males, HDL-C < 50mg/dL in females. Abnormal cardiovascular system: SBP ≥ 140 mmHg or DBP ≥ 90 mmHg, or self-reported hypertension or use of antihypertensive medication. * <0.05, ** <0.01.

**Figure 3:**
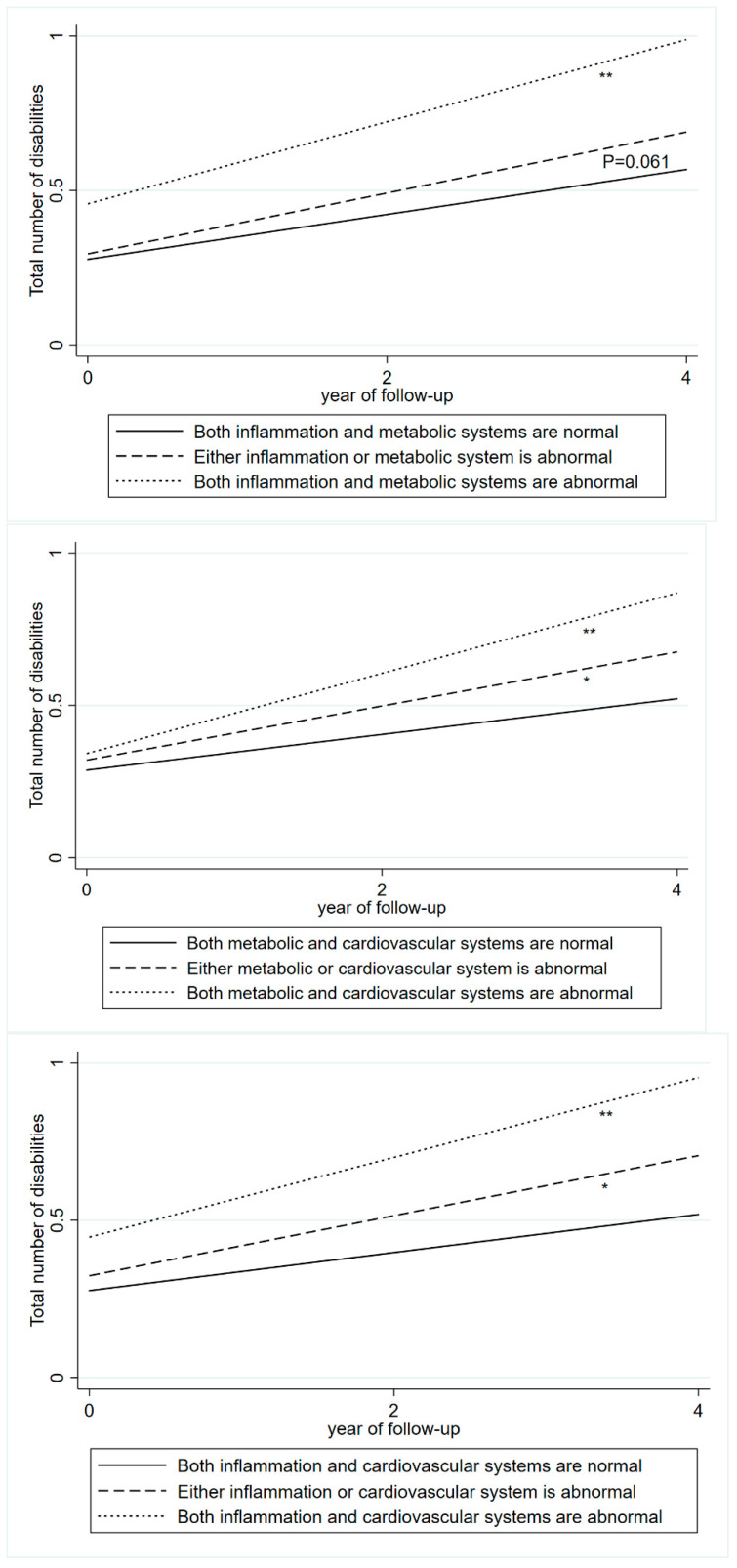
The cumulative burden of disabilities associated with the baseline abnormal levels of blood biomarkers in two biological systems. The cumulative burden was derived from a linear mixed-effects model adjusted for age, sex, education, marital status, smoking, alcohol consumption, BMI, health status, and depressive symptoms. Abnormal inflammation system: CRP > 3 mg/L. Abnormal metabolic system: HDL-C < 40 mg/dL in males, HDL-C < 50 mg/dL in females. Abnormal cardiovascular system: SBP ≥ 140 mmHg or DBP ≥ 90 mmHg, or self-reported hypertension or use of antihypertensive medication. * <0.05, ** <0.01.

**Figure 4:**
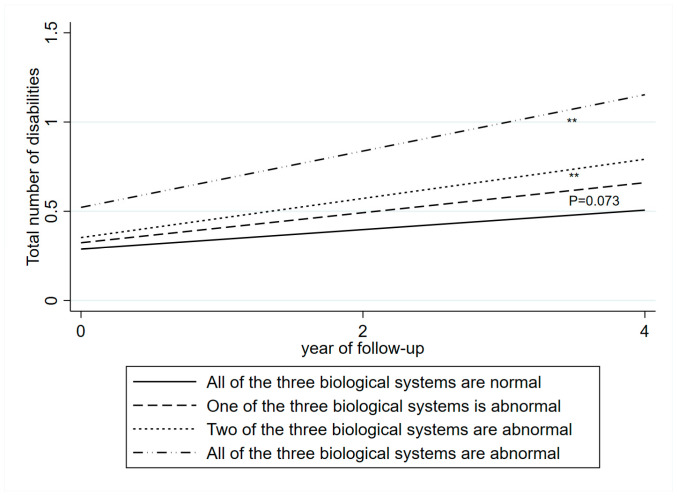
The cumulative burden of disabilities associated with the baseline abnormal levels of blood biomarkers in three biological systems. The cumulative burden was derived from linear mixed-effects model adjusted for age, sex, education, marital status, smoking, alcohol consumption, BMI, health status, and depressive symptom. Three abnormal biological systems: including inflammation system: CRP > 3 mg/L; metabolic system: HDL-C < 40 mg/dL in males, HDL-C < 50 mg/dL in females; cardiovascular system: SBP ≥ 140 mmHg or DBP ≥ 90 mmHg, or self-reported hypertension or use of antihypertensive medication. ** <0.01.

The cumulative burden of the total number of disabilities over 4 years in relation to abnormal levels of blood biomarkers across different biological systems is shown in Figure 2, Figure 3 and Figure 4. Abnormal levels of inflammation were associated with a steeper slope in the increase in the number of disabilities (β = 0.03). Similar trends were found for the metabolic (β = 0.03), and cardiovascular (β = 0.04) systems (Figure 2).

The cumulative burden of disabilities in individuals with abnormal levels of blood biomarkers in two biological systems is shown in Figure 3. A steeper increase in disability burden was associated with abnormal levels of blood biomarkers in both the inflammation and metabolic systems (β = 0.06), the metabolic and cardiovascular systems (β = 0.07), and the inflammation and cardiovascular systems (β = 0.07), compared with normal biomarker levels.

A clear dose–response association was observed. Compared with individuals with normal biomarker levels across all three systems, those with abnormal levels in any two systems (β = 0.06) or in all three systems (β = 0.1) experienced a significantly faster rate of disability accumulation over a 4-year period (Figure 4).

Stratified analyses by education revealed that abnormal levels of blood biomarkers in two and three biological systems were more strongly associated with faster disability accumulation among individuals with low education (≤9 years) than among those with higher education (>9 years) (0.09 vs. 0.04, 0.15 vs. 0.08).

### 3.3. Sensitivity Analysis

The associations between the presence of abnormal levels of blood biomarkers in pathophysiological or disease conditions and incidence of disability accumulation remained consistent across all sensitivity analyses: among those without disability, when using alternative clinical cutoffs, when including individuals with missing values in all biological systems that were replaced by imputation (Table S2). Notably, sensitivity analyses conducted separately in the HRS and CHARLS cohorts confirmed a consistent dose–response relationship (Table S3), indicating that the observed association was robust and not an artifact of pooling data collected under different measurement protocols.

## 4. Discussion

The present study revealed a dose–response relationship between the number of affected biological systems and the rate of disability accumulation, indicating that disability accumulation increases with the number of affected systems. These findings, together with the longitudinal design, underscore the clinical importance of monitoring blood biomarker levels in the inflammation, metabolic, and cardiovascular systems to identify individuals at higher risk of developing disability.

### 4.1. Comparison with Previous Studies

Our study builds upon previous findings by investigating both U.S. and Chinese cohorts using established clinical cutoffs to define abnormal levels of blood biomarkers, rather than relying on population-based percentiles as in earlier research [15,18,19]. A recent CHARLS-based study [33] also confirmed this association but used a larger biomarker panel and focused only on the Chinese population. Additionally, we extended previous findings by demonstrating a dose–response relationship between the number of affected biological systems and the rate of disability accumulation, supporting a potential causal association. Furthermore, we expanded these findings by performing stratified analysis by education level. The observation that lower education may amplify the adverse effect of abnormal biomarker levels on disabilities is consistent with prior evidence [34], which documented the synergistic effect of low education and high allostatic load on cancer mortality. This consistency across distinct outcomes highlights the fundamental role of education in buffering physiological and functional decline.

### 4.2. Methodological Considerations and Cohort Differences

A previous study proposed that DBS-based assays are appropriate for large cohort studies when relative rather than absolute values are used. That study reported that the values of CRP from DBS were highly correlated with venous CRP levels, whereas HDL-C values were less reliable [28]. This discrepancy may result from differences in the sensitivities of the fluorometric and enzymatic colorimetric methods, as well as potential influence of sampling methods, shipping time, temperature and humidity [35]. To address this issue, we repeated the main analysis within each cohort separately, and similar trends were observed (Table S3). Notably, individuals with abnormal levels in all three biological systems in CHARLS exhibited a significantly highly cumulative disability burden compare with those in HRS (0.17 vs. 0.07) (Table S3). This discrepancy could be explained by the fact that Chinese participants had higher mean SBP (Chinese: 148 mm Hg vs. U.S.: 133.3 mm Hg) and greater proportion with severe hypertension (≥160/100 mm Hg) (28.9% versus 11.6%), lower mean HDL-C levels (36.4 mg/dL vs. 39.1 mg/dL), and a higher proportion with very low level of HDL-C(<35 mg/dL) (39.5% vs. 25%) compared with U.S. participants. These findings align with a previous study comparing results from American and Chinese cohorts [36].

### 4.3. The Modifying Role of Education

The stronger association between multisystem dysregulation and disability accumulation among older adults with lower levels of education (≤9 years) suggests that socioeconomic resources, as reflected by educational attainment, may buffer physiological pathway leading to functional decline. This educational gradient could be explained by several factors: higher health literacy, enabling better self-management of chronic conditions; greater healthcare access, facilitating early detection and treatment of risk factors; and socioeconomic advantages that reduce allostatic load. From a clinical perspective, these findings suggest that adults ≥50 years with lower levels of education represent a high-risk group that could benefit most from targeted monitoring of routine biomarkers such as CRP, HDL-C, and blood pressure. Furthermore, integrating health education and self-management training into prevention programs could help mitigate the disproportionate disability risk in this vulnerable population.

### 4.4. Possible Mechanisms

The associations between abnormal biomarker levels and disability accumulation can be interpreted within the AL framework, which posits that chronic stress induces multisystem physiological dysregulation across inflammatory, metabolic, and cardiovascular systems [8,9]. Sustained stress responses over time lead to secondary alterations, elevated CRP indicating inflammation, reduced HDL-C reflecting metabolic dysfunction, and hypertension signifying cardiovascular strain, which collectively accelerate tertiary outcomes such as physical decline and disability [37].

Specifically, elevated CRP is associated with subclinical cerebrovascular disease and diffuse atherosclerosis, both of which impair physical function and contribute to frailty [2]. Reduced HDL-C diminishes vascular protection by impairing cholesterol efflux and anti-inflammatory capacity, promoting atherosclerotic progression and metabolic syndrome [38]. Hypertension exacerbates vascular damage, white matter lesions, and muscle mass loss, thereby accelerating cognitive and functional decline [3]. The synergistic dysregulation of these systems reflects cumulative “wear and tear” and may biologically mediate the pathway from chronic stress exposure to disability accumulation [8,9,37].

### 4.5. Public Health and Clinical Implications

Our findings have important implications for preventive care. To translate these into practice, we propose a tiered approach to biomarker monitoring. In routine clinical settings, blood pressure and HDL-C (as part of a standard lipid panel) should be prioritized. These are already core components of cardiovascular risk assessment, widely available, low-cost, and familiar to clinicians. CRP testing can be used for additional risk stratification in individuals already identified with one abnormal system (e.g., isolated hypertension or low HDL-C) to identify those with cumulative, multisystem dysregulation who are at the highest risk.

In low-resource settings, this approach remains practical. Blood pressure measurement is non-invasive, inexpensive, and universally implementable. While HDL-C and CRP require laboratory assays, their inclusion in a minimal targeted panel is far more feasible than using large biomarker batteries. The key clinical strategy is the integrated interpretation of available biomarkers rather than simultaneous testing of all three. This strategy can efficiently identify high-risk older adults, particularly those with lower educational attainment, enabling the targeting of limited resources for more intensive lifestyle or pharmacological interventions to prevent disability.

### 4.6. Strengths and Limitations

The strengths of this study are as follows: First, the inclusion of a large sample size (*n* = 16,094) from both western and eastern countries provides more reliable results with greater precision, generalizability, and statistical power. Second, the use of blood biomarkers reduces self-report or recall bias and improves accuracy, reliability, and validity, making it easier to compare data across different studies and populations. Third, utilizing a minimal biomarker set to represent biological systems strikes a balance between scientific rigor, practicality, and cost-effectiveness. This approach ensures that our study is not only theoretically grounded but also feasible in real-world applications. Fourth, we are the first to explore the potential dose–response relationship between the number of biological systems with abnormal value of blood biomarkers and the rate of disability accumulation, supporting a plausible causal association. In addition, the use of easily obtainable and lost-cost secondary mediators (CRP, HDL-C, SBP, DBP) together with self-reported hypertension or use of anti-hypertension medication enhances the feasibility of replicating this approach in other large cohort studies.

There are several limitations. First, missing values on blood biomarkers may lead to selection bias. However, the results remained similar in the sensitivity analyses when missing values were replaced by multiple imputations. Second, although the core constructs of ADL and IADL disability were assessed in both cohorts, specific items differed slightly. While this could influence absolute disability prevalence, our analysis focused on the cumulative burden (total number of disabilities) and its rate of change over time within each cohort. This combined and validated scale is more sensitive to change [29] and less dependent on items composition than binary disabled/not disabled classification. Third, although our longitudinal design helps establish temporal order, reverse causation cannot be completely ruled out, as disability might also influence biomarker levels through changes in physical activity, dietary habits, or comorbidities. To minimize this possibility, we excluded participants with disability at baseline and focused on incident disability during follow-up (Table S2). Fourth, our study is observational and lacks experimental validation, which limits causal inference. Although we observed a dose–response relationship between multisystem biomarker abnormalities and disability accumulation, causality cannot be established. Unmeasured confounding factors, such as genetic predisposition, diet, socioeconomic status, or early-life exposures, may influence both biomarker profiles and disability risk. Future interventional studies are needed to determine whether modifying these biomarkers can reduce disability risk and establish causal relationships. Fifth, as noted in the methods, the use of different biomarker assessment protocols (DBS in HRS vs. venous blood in CHARLS) may introduce measurement non-comparability, particularly for HDL-C. However, our use of clinical cut-offs and consistent cohort-specific results support the robustness of our pooled analyses. Sixth, reporting bias might exist because ADL, IADL, and some covariates were self-reported. Nevertheless, blood biomarkers were objectively measured, meaning any bias would likely underestimate the observed associations. Finally, our operationalization of allostatic load was limited to a minimal biomarker set (CRP, HDL-C, and blood pressure). Primary biomarkers such as cortisol, DHEA-S, and catecholamines, which are central to the allostatic load framework, were unavailable in these datasets. Future research incorporating this measure would provide a more comprehensive understanding of the multisystem pathway linking chronic stress to functional decline.

## 5. Conclusions

In conclusion, this study suggests that abnormal blood biomarker levels in any two or all three systems (inflammation, metabolic, and cardiovascular) may serve as valuable preclinical indicators of disability development among adults aged 50 years and older. Routine monitoring of these biomarkers may assist healthcare professionals in identifying individuals at elevated risk and in guiding the design of feasible, targeted prevention strategies.

## Figures and Tables

**Table 1:** Baseline characteristics of the study participants in relation to the biological systems.

	HRS					CHARLS				
	Normal Levels of Blood Biomarkers in the Biological Systems (*n* = 1624)	Abnormal Levels of Blood Biomarkers in One of the Biological Systems (*n* = 3835)	Abnormal Levels of Blood Biomarkers in Two of the Biological Systems (*n* = 2931)	Abnormal Levels of Blood Biomarkers in all of the Biological Systems ^a^ (*n* = 860)	*p*	Normal Levels of Blood Biomarkers in the Biological Systems (*n* = 2088)	Abnormal Levels of Blood Biomarkers in One of the Biological Systems (*n* = 2842)	Abnormal Levels of Blood Biomarkers in Two of the Biological Systems (*n* = 1549)	Abnormal Levels of Blood Biomarkers in All of the Biological Systems (*n* = 365)	*p*
Age (years)	62.3 ± 9.4	65.7 ± 10.1	65.8 ± 10	64.6 ± 9.7	**<0.001**	61.1 ± 7.4	62.3 ± 8	62.6 ± 8.1	63.8 ± 8.2	**<0.001**
Female sex	901 (55.5)	2042 (53.3)	1851 (63.2)	663 (77.1)	**<0.001**	903 (43.3)	1449 (51)	985 (63.6)	244 (66.9)	**<0.001**
Education					**<0.001**					0.323
≤9 years	112 (6.9)	403 (10.6)	376 (12.9)	122 (14.3)		1891 (90.6)	2598 (91.5)	1428 (92.3)	331 (90.7)	
>9 years	1501 (93.1)	3414 (89.4)	2542 (87.1)	734 (85.8)		196 (9.4)	243 (8.6)	119 (7.7)	34 (9.3)	
Marital status					**<0.001**					**<0.001**
Married	1111 (68.5)	2319 (60.5)	1644 (56.1)	437 (50.8)		1819 (87.1)	2347 (82.6)	1252 (80.8)	290 (79.5)	
Unmarried	512 (31.6)	1514 (39.5)	1287 (43.9)	423 (49.2)		269 (12.9)	495 (17.4)	297 (19.2)	75 (20.6)	
Smoking					**<0.001**					**<0.001**
Current smokers	207 (12.8)	514 (13.4)	481 (16.4)	177 (20.6)		772 (37)	883 (31.3)	359 (23.4)	73 (20.2)	
Non-current smokers	1417 (87.3)	3320 (86.6)	2450 (83.6)	682 (79.4)		1312 (63)	1938 (68.7)	1177 (76.6)	289 (79.8)	
Alcohol consumption					**<0.001**					**<0.001**
Regular alcohol drinkers	276 (17)	559 (14.6)	270 (9.2)	41 (4.8)		353 (18.2)	371 (13.8)	119 (8.1)	28 (7.9)	
Irregular alcohol drinkers	1347 (83)	3271 (85.4)	2656 (90.8)	817 (95.2)		1589 (81.8)	2316 (86.2)	1360 (92)	327 (92.1)	
BMI					**<0.001**					**<0.001**
Underweight (<18.5)	29 (1.8)	30 (0.8)	15 (0.6)	1 (0.2)		227 (11)	174 (6.5)	48 (3.4)	5 (1.5)	
Normal weight (18.5–24.99)	565 (35.9)	782 (21.9)	325 (13.3)	53 (8.6)		1544 (75.1)	1724 (64.7)	683 (48.8)	129 (38.7)	
Overweight (25–29.99)	607 (38.6)	1405 (39.3)	857 (35)	169 (27.5)		272 (13.2)	670 (25.1)	561 (40)	141 (42.3)	
Obese (≥30)	371 (23.6)	1354 (37.9)	1250 (51.1)	391 (63.7)		14 (0.7)	97 (3.6)	109 (7.8)	58 (17.4)	
Health status ^b^					**<0.001**					**<0.001**
Healthy	654 (40.5)	507 (13.3)	187 (6.4)	25 (2.9)		783 (39)	735 (27)	266 (17.8)	36 (10.3)	
Unhealthy	960 (59.5)	3300 (86.7)	2725 (93.6)	825 (97.1)		1223 (61)	1989 (73)	1229 (82.2)	313 (89.7)	
Depressive symptom	307 (19)	921 (24.1)	957 (32.9)	330 (38.8)	**<0.001**	734 (37.5)	1053 (39.7)	588 (41.2)	145 (43.4)	0.069
ADL disability	138 (8.5)	538 (14)	596 (20.3)	254 (29.5)	**<0.001**	332 (15.9)	539 (19)	319 (20.6)	91 (24.9)	**<0.001**
IADL disability	92 (5.7)	383 (10)	426 (14.5)	178 (20.7)	**<0.001**	399 (19.1)	665 (23.4)	415 (26.8)	111 (30.4)	**<0.001**

Notes: Data are presented as mean ± SD or n (%). HRS, Health and Retirement Study; CHARLS, China Health and Retirement Longitudinal Study; BMI, Body Mass Index; ADL, activities of daily living; IADL, instrumental ADL. ^a^ Biological systems: inflammation (CRP); metabolic (HDL-C); cardiovascular (SBP/DBP, self-reported hypertension, use of antihypertensive medication). ^b^ Healthy: no diseases were reported; Unhealthy: Had been diagnosed by a doctor with any disease.

## Data Availability

The data supporting this study’s findings are available from https://hrs.isr.umich.edu/data-products (HRS) and https://charls.charlsdata.com/pages/data/111/zh-cn.html (CHARLS).

## Supplementary Materials

The following supporting information can be downloaded at: https://www.mdpi.com/article/10.3390/jcdd12110429/s1, Table S1: Baseline characteristics of the study sample without and with missing values on blood biomarkers or/and disabilities in HRS or/and CHARLS; Table S2: Sensitivity analyses for the number of disabilities associated with abnormal levels of blood biomarkers in the biological systems; Table S3: The number of disabilities associated with abnormal levels of blood biomarkers in the biological systems by cohort; Figure S1: Proportion of individuals with abnormal/normal levels of blood biomarkers in the biological systems in the whole population (HRS+CHARLS). The completed STROBE checklist is available in the Supplementary Materials.

## Abbreviations

The following abbreviations are used in this manuscript:

ADL	Activities of daily living
AL	Allostatic load
ANOVA	Analysis of variance
BMI	Body mass index
CES-D	Center for Epidemiological Studies Depression scale
CHARLS	China Health and Retirement Longitudinal Study
China CDC	Chinese Center for Disease Control and Prevention
CRP	C-reactive protein
DBP	Diastolic blood pressure
DBS	Dried blood spot
ELISA	Enzyme-linked immunosorbent assay
HbA1c	Glycosylated hemoglobin
HDL-C	High-density lipoprotein cholesterol
HRS	Health and Retirement Study
IADL	Instrumental activities of daily living
SBP	Systolic blood pressure
