# Supplementary material for: Abnormal Blood Biomarkers and Cumulative Disability Burden in Middle-Aged and Older Adults: Evidence from Two Nationally Representative Surveys in the United States and China
Source: J Cardiovasc Dev Dis. 2025 Oct 31;12(11):429. doi: 10.3390/jcdd12110429 (PMC12653407; doi:10.3390/jcdd12110429)
Supplement: Supplementary file 1 [file jcdd-12-00429-s001.zip › jcdd-3896598-supplementary.pdf]

Journal of Cardiovascular Development and Disease

MDPI

*Supplementary Materials*

# Abnormal blood biomarkers and cumulative disability burden in middle-aged and older adults: evidence from two nationally representative surveys in the United States and China

**Raoping Tu [1]*, Jin-Jing Pei [2], Alexander Wolthon [2], Yueping Li [3] and Hui-Xin Wang [2]***

[1] School of Health Management, Fujian Medical University, 350000 Fuzhou, China; tototrp@126.com
[2] Division of Psychobiology and Epidemiology, Department of Psychology, Stockholm University, Stockholm, SE-106 91 Stockholm, Sweden; jin-jing.pei@su.se (J.J.P); alexander.wolthon@ki.se (A.W.); huixin.wang@su.se (Y.P.L.)
[3] Fujian Medical University Library, 350000 Fuzhou, Fujian, China; fmulyp@163.com
* Correspondence: tototrp@126.com, Tel.: +86-13489959482; huixin.wang@su.se, Tel.: +46- 08-553 789 08

**Table S1.** Baseline characteristics of the study sample without and with missing values on blood biomarkers or/and disabilities in HRS or/and CHARLS.

| | **HRS** | | | **CHARLS** | | | **HRS+CHARLS** | | |
|---|---|---|---|---|---|---|---|---|---|
| | **Non-missing (n=11176)** | **Missing (n=3847)** | ***p*** | **Non-missing (n=8196)** | **Missing (n=5646)** | ***p*** | **Non-missing (n=19372)** | **Missing (n= 9493)** | ***p*** |
| Age (years) | 65.8±10.5 | 68.4±11.3 | **<0.001** | 62.4±8.3 | 62.6±9.3 | 0.345 | 64.4±9.8 | 65±10.6 | **<0.001** |
| Female sex | 6478(58) | 2156(56.1) | **0.039** | 4237(51.7) | 2743(48.7) | **<0.001** | 10715(55.3) | 4899(51.7) | **<0.001** |
| Education | | | 0.065 | | | **<0.001** | | | **<0.001** |
| ≤9 years | 1287(11.6) | 486(12.7) | | 7456(91) | 4780(85.3) | | 8743(45.3) | 5266(55.8) | |
| >9 years | 9834(88.4) | 3343(87.3) | | 734(9) | 823(14.7) | | 10568(54.7) | 4166(44.2) | |
| Marital status | | | **0.001** | | | **<0.001** | | | 0.427 |
| Married | 6507(58.2) | 2356(61.3) | | 6722(82) | 4150(73.9) | | 13229(68.3) | 6506(68.8) | |
| Unmarried | 4665(41.8) | 1488(38.7) | | 1474(18) | 1467(26.1) | | 6139(31.7) | 2955(31.2) | |
| Smoking | | | **<0.001** | | | **0.028** | | | 0.817 |
| Current smokers | 1724(15.4) | 473(12.3) | | 2487(30.6) | 1466(28.8) | | 4211(21.8) | 1939(21.7) | |
| Non-current smokers | 9449(84.6) | 3373(87.7) | | 5649(69.4) | 3629(71.2) | | 15098(78.2) | 7002(78.3) | |
| Alcohol consumption | | | 0.228 | | | **0.001** | | | 0.111 |
| Regular alcohol drinkers | 1347(12.1) | 492(12.8) | | 1018(13.2) | 559(11.1) | | 2365(12.5) | 1051(11.8) | |
| Irregular alcohol drinkers | 9813(87.9) | 3349(87.2) | | 6720(86.8) | 4476(88.9) | | 16533(87.5) | 7825(88.2) | |
| BMI | | | 0.962 | | | **0.010** | | | **<0.001** |

| | **HRS** | | | **CHARLS** | | | **HRS+CHARLS** | | |
|---|---|---|---|---|---|---|---|---|---|
| | **Non-missing (n=11176)** | **Missing (n=3847)** | ***p*** | **Non-missing (n=8196)** | **Missing (n=5646)** | ***p*** | **Non-missing (n=19372)** | **Missing (n= 9493)** | ***p*** |

| | | | | | | | | | |
|---|---|---|---|---|---|---|---|---|---|
| Underweight (<18.5) | 105(1.1) | 37(1.1) | | 564(7.4) | 248(8.1) | | 669(3.8) | 285(4.5) | |
| Normal weight (18.5-24.99) | 2185(22.2) | 731(22.1) | | 4819(63) | 2013(65.4) | | 7004(40) | 2744(43) | |
| Overweight (25-29.99) | 3653(37.1) | 1241(37.5) | | 1938(25.4) | 688(22.3) | | 5591(31.9) | 1929(30.2) | |
| Obese (≥30) | 3915(39.7) | 1302(39.3) | | 325(4.3) | 130(4.2) | | 4240(24.2) | 1432(22.4) | |
| Health status[a] | | | **<0.001** | | | **<0.001** | | | **<0.001** |
| Healthy | 1583(14.3) | 439(11.6) | | 2109(26.8) | 1869(35.3) | | 3692(19.5) | 2308(25.4) | |
| Unhealthy | 9492(85.7) | 3347(88.4) | | 5752(73.2) | 3433(64.8) | | 15244(80.5) | 6780(74.6) | |
| Depressive symptom | 3165(28.5) | 1042(27.3) | 0.150 | 3052(40.2) | 1569(36.2) | **<0.001** | 6217(33.3) | 2611(32.1) | 0.054 |

[a] Health status: Healthy: no such report. Unhealthy: Had been diagnosed by a doctor with any chronic disease. HRS, Health and Retirement Study; CHARLS, China Health and Retirement Longitudinal Study; BMI, Body Mass Index.

**Table S2.** Sensitivity analyses for the number of disabilities associated with abnormal levels of blood biomarkers in the biological systems.

| | **Among those without disability at baseline (after excluding 4064 individuals with disability)** | | | **Using HDL-C<40mg/dL as cut-offs for metabolic system in combination with inflammation and cardiovascular systems** | | | **After imputation on individuals with missing data in all three biological systems** | | |
|---|---|---|---|---|---|---|---|---|---|
| | ***β*[a]** | **95% CI** | ***p*** | ***β*** | **95% CI** | ***p*** | ***β*** | **95% CI** | ***p*** |
| **Longitudinal association[b]** | | | | | | | | | |
| Inflammation system[c] x time | 0.03 | 0.01 to 0.06 | **0.015** | 0.030 | 0 to 0.06 | 0.050 | 0.020 | -0.01 to 0.05 | 0.136 |
| Metabolic system[d] x time | 0.04 | 0.01 to 0.06 | **0.002** | 0.040 | 0.02 to 0.07 | **0.001** | 0.050 | 0.02 to 0.07 | **<0.001** |
| Cardiovascular system[e] x time | 0.05 | 0.02 to 0.07 | **<0.001** | 0.040 | 0.01 to 0.08 | **0.006** | 0.030 | 0 to 0.05 | **0.042** |
| **Inflammation and metabolic systems x time** | | | | | | | | | |
| Normal levels of blood biomarkers in these two systems | | Ref. | | | Ref. | | | Ref. | |
| Abnormal levels of blood biomarkers in one of the systems | 0.03 | 0.01 to 0.06 | **0.006** | 0.040 | 0.01 to 0.06 | **0.008** | 0.020 | 0 to 0.05 | 0.103 |
| Abnormal levels of blood biomarkers in both of the systems | 0.08 | 0.04 to 0.13 | **<0.001** | 0.070 | 0.01 to 0.12 | **0.022** | 0.050 | 0.01 to 0.09 | **0.021** |
| **Metabolic and cardiovascular systems x time** | | | | | | | | | |
| Normal levels of blood biomarkers in these two systems | | Ref. | | | Ref. | | | Ref. | |
| Abnormal levels of blood biomarkers in one of the systems | 0.04 | 0.01 to 0.06 | **0.006** | 0.030 | 0 to 0.06 | **0.026** | 0.030 | 0 to 0.06 | **0.040** |

| | | | | | | | | | |
|---|---|---|---|---|---|---|---|---|---|
| Abnormal levels of blood biomarkers in both of the systems | 0.08 | 0.05 to 0.11 | **<0.001** | 0.090 | 0.05 to 0.14 | **<0.001** | 0.070 | 0.03 to 0.1 | **<0.001** |
| | **Among those without disability at baseline (after excluding 4064 individuals with disability)** | | | **Using HDL-C<40mg/dL as cut-offs for metabolic system in combination with inflammation and cardiovascular systems** | | | **After imputation on individuals with missing data in all three biological systems** | | |
| | ***β*** | **95% CI** | ***p*** | ***β*** | **95% CI** | ***p*** | ***β*** | **95% CI** | ***p*** |
| **Inflammation and cardiovascular systems x time** | | | | | | | | | |
| Normal levels of blood biomarkers in these two systems | | Ref. | | | Ref. | | | Ref. | |
| Abnormal levels of blood biomarkers in one of the systems | 0.04 | 0.02 to 0.07 | **0.001** | 0.030 | 0.01 to 0.06 | **0.015** | 0.030 | 0.01 to 0.06 | **0.015** |
| Abnormal levels of blood biomarkers in both of the systems | 0.06 | 0.02 to 0.09 | **0.001** | 0.070 | 0.03 to 0.1 | **0.001** | 0.060 | 0.02 to 0.09 | **0.002** |
| **Inflammation, metabolic and cardiovascular systems x time** | | | | | | | | | |
| Normal levels of blood biomarkers in these three systems | | Ref. | | | Ref. | | | Ref. | |
| Abnormal levels of blood biomarkers in one of the systems | 0.05 | 0.02 to 0.07 | **0.001** | 0.030 | 0 to 0.06 | **0.028** | 0.030 | -0.01 to 0.06 | 0.104 |
| Abnormal levels of blood biomarkers in two of the systems | 0.06 | 0.03 to 0.09 | **<0.001** | 0.060 | 0.03 to 0.1 | **0.001** | 0.050 | 0.02 to 0.08 | **0.005** |
| Abnormal levels of blood biomarkers in all of the systems | 0.13 | 0.08 to 0.18 | **<0.001** | 0.130 | 0.06 to 0.2 | **<0.001** | 0.090 | 0.04 to 0.15 | **0.001** |

[a] Adjusted for age, sex, education, marital status, smoking, alcohol consumption, BMI, health status, depressive symptom

[b] Association between baseline abnormal biological systems and annual rate of disability increase (change in the number of total disabilities) over 4 years

[c] Abnormal inflammation system: CRP>3mg/L

[d] Abnormal metabolic system: HDL-C<40mg/dL in males, HDL-C<50mg/dL in females

[e] Abnormal cardiovascular system: SBP≥140mmHg or DBP≥90mmHg, or self-reported hypertension or use of antihypertensive medication

Ref., reference group.

a Adjusted for age, sex, education, marital status, smoking, alcohol consumption, BMI, health status, depressive symptom. b Association between baseline abnormal biological systems and annual rate of disability increase (change in the number of total disabilities) over 4 years. c Abnormal inflammation system: CRP>3mg/L. d Abnormal metabolic system: HDL-C<40mg/dL in males, HDL-C<50mg/dL in females. e Abnormal cardiovascular system: SBP≥140mmHg or DBP≥90mmHg, or self-reported hypertension or use of antihypertensive medication. Ref., reference group.

**Table S3.** The number of disabilities associated with abnormal levels of blood biomarkers in the biological systems by cohort.

| Inflammation[a], metabolic[b] and cardiovascular[c] systems x time[d] | HRS+CHARLS | | | | HRS | | | | CHARLS | | | |
|---|---|---|---|---|---|---|---|---|---|---|---|---|
| | N | $\beta^e$ | 95% CI | $p$ | N | $\beta$ | 95% CI | $p$ | N | $\beta$ | 95% CI | $p$ |
| Normal levels of blood biomarkers in these three systems | 3712 | | Ref. | | 1624 | | Ref. | | 2088 | | Ref. | |
| Abnormal levels of blood biomarkers in one of the systems | 6677 | 0.03 | 0 to 0.06 | 0.073 | 3835 | 0.04 | 0 to 0.08 | **0.026** | 2842 | 0.02 | -0.04 to 0.07 | 0.496 |
| Abnormal levels of blood biomarkers in two of the systems | 4480 | 0.06 | 0.02 to 0.09 | **0.002** | 2931 | 0.06 | 0.02 to 0.1 | **0.006** | 1549 | 0.07 | 0 to 0.13 | **0.046** |
| Abnormal levels of blood biomarkers in all of the systems | 1225 | 0.1 | 0.05 to 0.16 | **<0.001** | 860 | 0.07 | 0.02 to 0.13 | **0.014** | 365 | 0.17 | 0.06 to 0.28 | **0.002** |

[a] Abnormal inflammation system: CRP>3mg/L

[b] Abnormal metabolic system: HDL-C<40mg/dL in males, HDL-C<50mg/dL in females

[c] Abnormal cardiovascular system: SBP≥140mmHg or DBP≥90mmHg, or self-reported hypertension or use of antihypertensive medication

[d] Association between baseline abnormal biological systems and annual rate of disability increase (change in the total number of disabilities) over 4 years

[e] Adjusted for age, sex, education, marital status, smoking, alcohol consumption, BMI, health status, depressive symptoms

Ref., reference group.

a Abnormal inflammation system: CRP>3mg/L. b Abnormal metabolic system: HDL-C<40mg/dL in males, HDL-C<50mg/dL in females. c Abnormal cardiovascular system: SBP≥140mmHg or DBP≥90mmHg, or self-reported hypertension or use of antihypertensive medication. d Association between baseline abnormal biological systems and annual rate of disability increase (change in the total number of disabilities) over 4 years. e Adjusted for age, sex, education, marital status, smoking, alcohol consumption, BMI, health status, depressive symptoms. Ref., reference group.

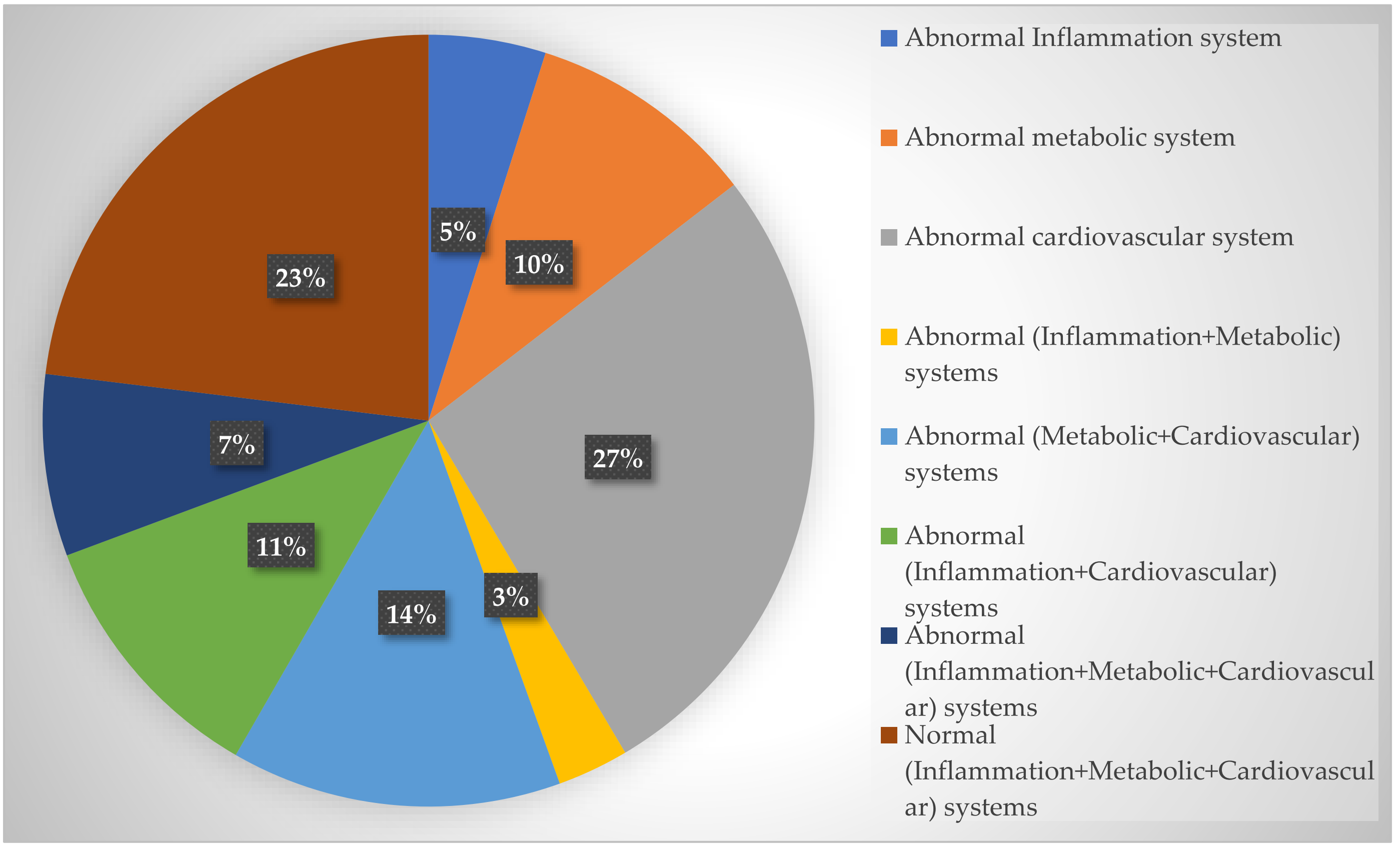


**Figure S1.** Proportion of individuals with abnormal/normal levels of blood biomarkers in the biological systems in the whole population (HRS+CHARLS). Abnormal inflammation system: CRP>3mg/L. Abnormal metabolic system: HDL-C<40mg/dL in males, HDL-C<50mg/dL in females. Abnormal cardiovascular system: SBP≥140mmHg or DBP≥90mmHg, or self-reported hypertension or use of antihypertensive medication.

Journal of Cardiovascular Development and Disease

MDPI

STROBE Statement—checklist of items that should be included in reports of observational studies

| | Item No. | Recommendation | Page No. | Relevant text from manuscript |
|---|---|---|---|---|
| **Title and abstract** | 1 | (*a*) Indicate the study's design with a commonly used term in the title or the abstract | P1 | Line 16-18 |
| | | (*b*) Provide in the abstract an informative and balanced summary of what was done and what was found | P1 | Line 12-33 |
| **Introduction** | | | | |
| Background/rationale | 2 | Explain the scientific background and rationale for the investigation being reported | P1-2 | Line 36-75 |
| Objectives | 3 | State specific objectives, including any prespecified hypotheses | P2 | Line 76-80 |
| **Methods** | | | | |
| Study design | 4 | Present key elements of study design early in the paper | P2-3 | Line 83-87, 91-94 |
| Setting | 5 | Describe the setting, locations, and relevant dates, including periods of recruitment, exposure, follow-up, and data collection | P2-3 | Line 83-90, 91-99 |
| Participants | 6 | (*a*) *Cohort study*—Give the eligibility criteria, and the sources and methods of selection of participants. Describe methods of follow-up<br>*Case-control study*—Give the eligibility criteria, and the sources and methods of case ascertainment and control selection. Give the rationale for the choice of cases and controls<br>*Cross-sectional study*—Give the eligibility criteria, and the sources and methods of selection of participants | P2-3 | Line 83-90, 91-99 |
| | | (*b*) *Cohort study*—For matched studies, give matching criteria and number of exposed and unexposed | Not applicable | Not applicable |

| | | | | |
|---|---|---|---|---|
| | | *Case-control study*—For matched studies, give matching criteria and the number of controls per case | | |
| Variables | 7 | Clearly define all outcomes, exposures, predictors, potential confounders, and effect modifiers. Give diagnostic criteria, if applicable | P3 | Line 100-136 |
| Data sources/ measurement | 8* | For each variable of interest, give sources of data and details of methods of assessment (measurement). Describe comparability of assessment methods if there is more than one group | P3 | Line 100-136 |
| Bias | 9 | Describe any efforts to address potential sources of bias | P4 | Line 154-160 |
| Study size | 10 | Explain how the study size was arrived at | P2-3, P14 | Line 83-99, Figure 1 |

Continued on next page

| | | | | |
|---|---|---|---|---|
| Quantitative variables | 11 | Explain how quantitative variables were handled in the analyses. If applicable, describe which groupings were chosen and why | P3 | Line 100-136 |
| Statistical methods | 12 | (*a*) Describe all statistical methods, including those used to control for confounding | P3-4 | Line 137-153 |
| | | (*b*) Describe any methods used to examine subgroups and interactions | P3 | Line 137-140 |
| | | (*c*) Explain how missing data were addressed | P4 | Line 158-159 |
| | | (*d*) *Cohort study*—If applicable, explain how loss to follow-up was addressed<br>*Case-control study*—If applicable, explain how matching of cases and controls was addressed<br>*Cross-sectional study*—If applicable, describe analytical methods taking account of sampling strategy | P4, P19-20 | Line 158-159, 172-174, Table S1 |
| | | (*e*) Describe any sensitivity analyses | P4 | Line 154-160 |
| **Results** | | | | |
| Participants | 13* | (a) Report numbers of individuals at each stage of study—eg numbers potentially eligible, examined for eligibility, confirmed eligible, included in the study, completing follow-up, and analysed | P4 | Line 163-170, Figure 1 |
| | | (b) Give reasons for non-participation at each stage | P14 | Figure 1 |
| | | (c) Consider use of a flow diagram | P14 | Figure 1 |
| Descriptive data | 14* | (a) Give characteristics of study participants (eg demographic, clinical, social) and information on exposures and potential confounders | P4 | Line 163-178 |
| | | (b) Indicate number of participants with missing data for each variable of interest | P4, P19 | Line 172-174, Table S1 |
| | | (c) *Cohort study*—Summarise follow-up time (eg, average and total amount) | P14 | Figure 1 |
| Outcome data | 15* | *Cohort study*—Report numbers of outcome events or summary measures over time | P11-13 | Table 1 |
| | | *Case-control study*—Report numbers in each exposure category, or summary measures of exposure | Not applicable | Not applicable |
| | | *Cross-sectional study*—Report numbers of outcome events or summary measures | Not applicable | Not applicable |
| Main results | 16 | (*a*) Give unadjusted estimates and, if applicable, confounder-adjusted estimates and their precision (eg, 95% confidence interval). Make clear which confounders were adjusted for and why they were included | P4-5, P15-18 | Line 179-199, Figure 2-4 |

| | | | |
|---|---|---|---|
| | (*b*) Report category boundaries when continuous variables were categorized | P3 | Line 100-136 |
| | (*c*) If relevant, consider translating estimates of relative risk into absolute risk for a meaningful time period | Not applicable | Not applicable |

Continued on next page

| | | | | |
|---|---|---|---|---|
| Other analyses | 17 | Report other analyses done—eg analyses of subgroups and interactions, and sensitivity analyses | P5, P21-23 | Line 200-206, Table S2, S3 |
| **Discussion** | | | | |
| Key results | 18 | Summarise key results with reference to study objectives | P5 | Line 208-213 |
| Limitations | 19 | Discuss limitations of the study, taking into account sources of potential bias or imprecision. Discuss both direction and magnitude of any potential bias | P7 | Line 297-308 |
| Interpretation | 20 | Give a cautious overall interpretation of results considering objectives, limitations, multiplicity of analyses, results from similar studies, and other relevant evidence | P5-6 | Line 214-281 |
| Generalisability | 21 | Discuss the generalisability (external validity) of the study results | P6-7 | Line 282-296 |
| **Other information** | | | | |
| Funding | 22 | Give the source of funding and the role of the funders for the present study and, if applicable, for the original study on which the present article is based | P7 | Line 329-335 |

*Give information separately for cases and controls in case-control studies and, if applicable, for exposed and unexposed groups in cohort and cross-sectional studies.

**Note:** An Explanation and Elaboration article discusses each checklist item and gives methodological background and published examples of transparent reporting. The STROBE checklist is best used in conjunction with this article (freely available on the Web sites of PLoS Medicine at http://www.plosmedicine.org/, Annals of Internal Medicine at http://www.annals.org/, and Epidemiology at http://www.epidem.com/). Information on the STROBE Initiative is available at www.strobe-statement.org.
